# Efficacy of temsirolimus in metastatic chromophobe renal cell carcinoma

**DOI:** 10.1186/1471-2490-13-26

**Published:** 2013-05-21

**Authors:** Balaji Venugopal, Jawaher Ansari, Michael Aitchison, Lye Mun Tho, Roderick Campbell, Rob J Jones

**Affiliations:** 1 Beatson West of Scotland Cancer Centre, 1053, Great Western Road, Glasgow, G12 0YN, UK; 2 Institute of Cancer Sciences, University of Glasgow, Glasgow, G12 8QQ, UK; 3 Monklands Hospital, Monkscourt Avenue, Airdrie, ML6 0JS, UK

**Keywords:** Temsirolimus, Chromophobe renal cell carcinoma, Renal cell cancer

## Abstract

**Background:**

Renal cell carcinoma (RCC) is a histopathologically and molecularly heterogeneous disease with the chromophobe subtype (chRCC) accounting for approximately 5% of all cases. The median overall survival of advanced RCC has improved significantly since the advent of tyrosine kinase inhibitors and mammalian target of rapamycin (mTOR) inhibitors. However, high-quality evidence for the use of new generation tyrosine kinase inhibitors in patients with advanced chRCC is lacking. Few published case reports have highlighted the use of temsirolimus in chRCC.

**Case presentation:**

Here, we report the case of a 36-year-old Caucasian woman with metastatic chRCC with predominantly skeletal metastases who was refractory to sunitinib who demonstrated a durable clinical response to temsirolimus lasting 20 months. We review the available evidence pertaining to the use of new generation molecularly targeted agents, in particular mTOR inhibitors in chRCC and discuss their emerging role in the management of this disease which would aid the oncologists faced with the challenge of treating this rare type of RCC.

**Conclusion:**

Conducting randomised clinical trials in this rarer sub-group of patients would be challenging and our case report and the evidence reviewed would guide the physicians to make informed decision regarding the management of these patients.

## Background

Renal cell carcinoma (RCC) accounts for 2-3% of all malignancies and is the seventh most common malignancy in men and the twelfth most common malignancy in women [1]. Molecularly targeted agents inhibiting the angiogenic and mTOR pathways have widened the therapeutic armamentarium for RCC and have led to a paradigm shift in the management of this disease particularly in the metastatic setting [2]. RCC is a heterogeneous disease characterised by distinct histological subtypes, molecular genetic alterations, clinical behaviour and patient outcomes, and the subtypes include clear cell (70-80%), papillary (10-20%), chromophobe (5%), collecting duct (1%) and unclassifiable RCC [3].

Histological appearances of chRCC typically demonstrate aggregates of pale cells with granular to eosinophilic cytoplasm and prominent cell membranes. Nuclear features are particularly useful in making the diagnosis, and distinguishing chromophobe carcinoma from other forms of renal carcinoma and oncocytoma. The nuclei in chromophobe carcinomas are generally dark and wrinkled, with a surrounding peri-nuclear halo of clear cytoplasm. Ancillary studies can also be helpful particularly if morphology is indeterminate. Staining for Hale’s colloidal iron is often positive and immunohistochemical markers are usually negative for cytokeratin 20 and vimentin but positive for cytokeratin 7 [4,5].

We report a case of a patient with a diagnosis of metastatic chromophobe renal cell carcinoma that was refractory to treatment with sunitinib but achieved durable clinical response lasting twenty months upon treatment with temsirolimus.

## Case presentation

**Figure 1:**
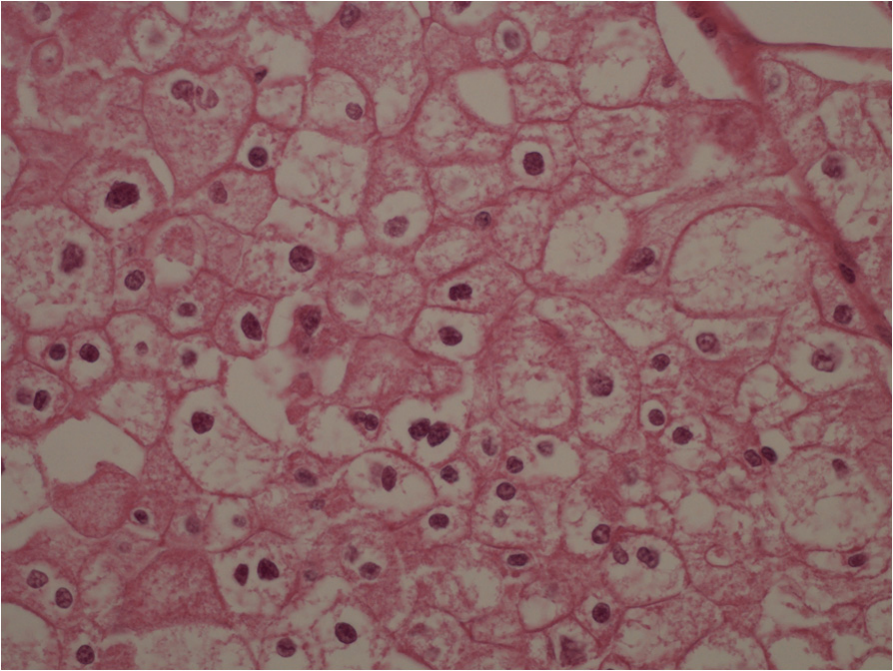
**Histology of chromophobe renal cell carcinoma.** Hematoxylin and eosin stained slide of the of nephrectomy specimen showing typical features of chromophobe renal cell carcinoma at original magnification times 400.

A 36-year-old woman was admitted to the emergency department with 6 months history of left sided back pain. Subsequent computed tomography (CT) scan and magnetic resonance imaging (MRI) revealed a complex 3 cm mass in left kidney, solitary para-aortic lymphadenopathy and osteolytic lesions within the thoracic and lumbar vertebrae. Following this, a CT guided biopsy of the renal mass was undertaken but this failed to provide definitive histological diagnosis. Due to bony disease causing impending spinal cord compression at thoracic vertebrae T12 (without neurological deficit), she received urgent radiotherapy (20Grays in 5 fractions) to this area which did not result in any significant improvement in her performance status. Following this, the decision was taken to proceed to a left laparoscopic cytoreductive nephrectomy and histopathological examination confirmed this to be a chRCC (Figure 1) with final staging of pT3a, pN1, M1 (as per American Joint Committee for Staging Cancer version 7).

Our patient presented with 4 of the six adverse prognostic factors as defined in the pivotal trial of Hudes et al. (haemoglobin less than lower limit of normal, Karnofsky performance status score of 70% and disease requiring systemic treatment within a year of presentation), thus categorising her within the poor prognostic grouping [6]. Following an uneventful post-operative recovery period, in January 2010 the patient was commenced on sunitinib at the recommended dose of 50 milligrams (mg) once daily for four weeks followed by two weeks off treatment. The dose was reduced to 37.5 mg once daily after cycle 1 due to persistent grade III thrombocytopaenia. After 2 cycles of sunitinib, there was clear clinical progression with deterioration of symptoms. CT and isotope bone scan demonstrated increase in size and number of bony metastases and sunitinib treatment was therefore discontinued. Sunitinib although is licenced for the use of both clear cell and non-clear RCC, the pivotal trial reported by Motzer and colleagues had excluded patients with non-clear cell RCC [7].

**Figure 2:**
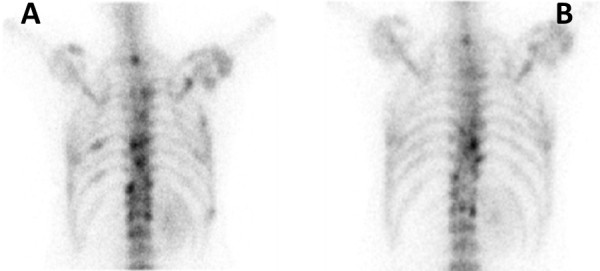
**Sequential isotope bone scans of the patient.** Isotope bone scans demonstrating response to treatment with reduction in size and number of skeletal metastases when compared to baseline (left panel, **A**) and 3 months after treatment (right panel, **B**) with temsirolimus.

At this point, the patient required physical aids to mobilise due to painful lytic bony metastases of the femur. In view of the non-clear histology and the adverse prognostic factor at diagnosis, in May 2010 the decision was then taken to commence temsirolimus at the recommended dose of 25 mg administered as weekly intravenous infusion. Treatment response was monitored using serial CT and isotope bone scans. Following 8 weeks of temsirolimus, there was significant improvement in pain control. Mobility was significantly better and the patient was able to mobilise independently without aid. Bone scan at baseline (Figure 2, panel A) when compared with the bone scan after three months of treatment with temsirolimus demonstrated reduced isotope uptake in the metastatic lesions (Figure 2, panel B). CT scans showed features of stable disease without the appearance of any new lesions. Taken together this demonstrated a favourable response to treatment. Toxicities of temsirolimus were minimal, which included grade I nail changes and grade I lethargy. She also received zolendronic acid at the dose of 4 mg as 4-weekly intravenous infusions from three months into treatment with temsirolimus.

Following 13 months of treatment with temsirolimus the patient developed acute onset dyspnoea on exertion. CT scan of the thorax revealed consolidation and interstitial changes of both left upper and right lower lobes of the lungs. A diagnosis of temsirolimus induced pneumonitis was made based on clinical and radiological grounds. Temsirolimus was withheld and antibiotics and steroids were given. Over a period of 2 weeks, the patient responded very well to this therapy and a chest X-ray (CXR) demonstrated significant resolution of the consolidative changes. Temsirolimus was re-commenced but within 2 weeks further dyspnoea re-emerged. The patient was treated with steroids and temsirolimus was withheld again. Following resolution of dyspnoea treatment was re-started at a reduced dose of temsirolimus (20 mg weekly). Following a further 2 months of reduced dose treatment, in January 2012, radiological evidence of disease progression with multiple, new hepatic metastases was apparent, at which point treatment with temsirolimus was discontinued. The patient died of progressive disease in April 2012, 26 months after diagnosis. In conclusion, we report a case of metastatic chRCC deriving significant clinical benefit from temsirolimus 20 months in duration, in the absence of prior response to sunitinib.

## Discussion

The prognosis of RCC varies significantly depending on histological sub-type, with non-clear cell histology portending a favourable prognosis compared with clear cell RCC [5,8]. Amongst the non-clear cell variety, patients with chRCC demonstrate significantly higher median overall survival compared to both clear cell and papillary RCC. Besides the histology, tumour stage, tumour grade and performance status are also independent prognostic markers of survival [5]. However these data were primarily derived prior to the advent of new generation targeted agents. Conversely, in the metastatic setting, non-clear cell RCCs are in general characterised by resistance to systemic therapy and poor survival [5,8]. In clinical trials of systemic therapy in the metastatic RCC, chRCC are continually under-represented and usually systematically excluded and it is difficult to draw conclusions to guide management decisions. This is particularly relevant in the era of modern targeted therapies, where the efficacy of newer agents such as sunitinib and temsirolimus in chRCC treatment remains uncertain.

The exclusion of non-clear cell RCC from clinical trials may be driven our limited and patchy understanding of the molecular biology of RCC. Abrogation of tumour suppressor function of the Von Hippel-Lindau (VHL) gene is a common feature of clear cell RCC, whereas the driving mutations behind non-clear cell RCC carcinogenesis are less well understood [9]. This has led to most contemporary clinical trials in RCC excluding patients with non-clear cell histology leading to a poor evidence base for this disease. However, the pivotal phase III clinical trial comparing temsirolimus versus interferon alpha (IFN) in metastatic RCC with at least three adverse poor prognostic features by Hudes and colleagues did include approximately 18% (n=37) of patients with non-clear cell RCC [6]. In this trial, there was only one patient with chRCC but the outcome of this specific patient is unknown [10]. In a subgroup analysis, the median overall survival was comparable for patients with clear cell and non-clear cell histology when treated with temsirolimus (10.7 months versus 11.6 months), whereas patients with clear cell and non-clear cell histology treated with IFN had poorer median OS (8.2 months versus 4.3 months) [10]. It appears that although temsirolimus demonstrated significant anti-tumour activity across all histological subtypes, the differential gain in efficacy of temsirolimus versus IFN seemed to be greatest in non-clear cell RCC. With the caveats of over-interpretation of data derived from retrospective subgroup analysis and the small numbers of patient involved, these data suggest that temsirolimus may have beneficial activity against non-clear cell RCC.

Choueiri et al. reported on sunitinib and sorafenib as first line therapy for metastatic papillary and chromophobe RCC in a series of 53 patients gathered from 4 centres in France and one in the USA [11]. Of the 12 patients with metastatic chRCC, only three had a partial response (two patients treated with sorafenib and one treated with sunitinib). This suggests that VEGF targeted tyrosine kinase inhibitors may only have modest activity against chRCC. This data, however, should be interpreted with caution in view of the small sample size.

Sporadic case reports exist in the literature documenting disease response of chRCC to mTOR inhibitors that have licenced for use in RCC, namely everolimus and temsirolimus. Larkin and colleagues have reported a case of a patient with chRCC with an ongoing 24 month period of disease response to everolimus as a second line treatment, following initial treatment with sunitinib [12]. Paule et al. report a case of chRCC responding to temsirolimus after initial treatment with interferon alpha and sorafenib [13]. Another case report also describes response to temsirolimus in a patient with metastatic chRCC who had initially responded to sunitinib and sorafenib [14]. A review of Surveillance Epidemiology and End Result (SEER)-17 program indicated that the deaths attributed to chRCC was between 4–9 cases per year and this accounted for 1% of total mortality rate due to RCC [15]. This report by Shuch and colleagues emphasize the rarity of chRCC and the challenges faced by clinicians in treating such rare tumours.

A prospective randomised clinical trial of first-line sunitinib versus everolimus in patients with metastatic non-clear cell RCC (ASPEN) is currently recruiting patients in the United Kingdom and North America [16]. The results of this trial are eagerly awaited, including implications for chRCC management in particular. The ideal scenario would be if the oncology community could perform adequately powered randomised clinical trials for chRCC specifically; however this would be fraught with various challenges involved in conducting clinical trials for rarer tumour types.

## Conclusion

In conclusion, there is insufficient evidence to produce definitive treatment recommendations for metastatic chRCC. Nevertheless there is an emerging theme that mTOR inhibitors are important in the management of this disease. Our case report and data reviewed herewith would indicate that temsirolimus is a reasonable first line treatment choice in the metastatic chRCC.

### Consent

Written informed consent was obtained from the patient for publication of this case report and any accompanying images. A copy of the written consent is available for review by the Series Editor of this journal.

## Competing interests

BV, JA, MA, RC, LM T declare that they have no competing interest. RJJ: Research funding and paid consultancy from Pfizer.

## Authors’ contributions

BV obtained the consent from the patient, wrote the manuscript and made the revisions. RC wrote the section on histology and provided the slide for histology. JA, MA, LM T and RJJ critically reviewed and amended the manuscript. All authors read and approved the final manuscript.

## Pre-publication history

The pre-publication history for this paper can be accessed here:

http://www.biomedcentral.com/1471-2490/13/26/prepub
